# The evolution of time use approaches for understanding activities of daily living in a public health context

**DOI:** 10.1186/s12889-019-6759-4

**Published:** 2019-06-03

**Authors:** Josephine Y. Chau, Sjaan R. Gomersall, Hidde P. van der Ploeg, Karen Milton

**Affiliations:** 1 Department of Health Systems and Populations, Faculty of Medicine and Health Sciences, Macquarie University, Sydney, Australia; 2 Prevention Research Collaboration, Sydney School of Public Health and Charles Perkins Centre, University of Sydney, Sydney, Australia; 3 School of Health and Rehabilitation Sciences, The University of Queensland, Brisbane, Australia; 4 Department of Public and Occupational Health, Amsterdam Public Health Research Institute, Amsterdam UMC, Vrije Universiteit Amsterdam, Amsterdam, the Netherlands; 5 Norwich Medical School, University of East Anglia, Norwich, England

**Keywords:** Time use, Physical activity, Sedentary behaviour, Diet, Health

## Abstract

This Supplement aims to raise awareness and knowledge of how time use surveys may be applied to studying health behaviours such as physical activity, sedentary behaviour, and eating. This commentary provides an overview and discussion of the papers in this Supplement about time use and health research, and considers possible future directions for the field.

## Introduction

Originally conceived by sociologists and economists, time use surveys provide a rich source of data about time spent in different activities of daily living (e.g., sleeping, eating, exercising, working, travelling) and how patterns of time use vary across demographic groups, between countries, and over time. This *BMC Public Health* supplement is a multidisciplinary endeavour, which brings together researchers from public health and sociology to showcase developments in the application of time use methods for studying health behaviours, focusing on the spectrum of physical behaviours such as sleep, sedentary behaviour, physical activity and diet.

Research about time use or time budgeting has been conducted since the 1920s [1], consequently, the concept of 24-h data detailing how people use their time is not new. However, it is only relatively recently that this approach has been adapted and applied in the public health context to examine patterns of time use and behaviours over a 24-h day, as Professors Bauman, Gershuny and Bittman highlight in their review of time use research and its relevance to public health. Advantages of time use diaries include minimising recall bias and reducing reporting errors, which are common to self-report, aggregate style questionnaires when participants are asked to estimate weekly or daily time spent in sleep, sedentary behaviour and physical activity.

In this supplement, we explore advances in the application of time use approaches for studying physical activity, sedentary behaviour, and eating behaviours. Broadly, the studies included herein cover three themes: using ‘traditional’ time use surveys in behavioural epidemiology; innovations in time use methods and data analysis; and the application of time use methods in observational and intervention studies.

### Harnessing ‘traditional’ time use surveys for behavioural epidemiology

National labour or statistics agencies typically use ‘traditional’ time use diaries to capture participants’ daily activities in set intervals (5–15 min) for 24 or 48 h, along with contextual details such as where, and with whom, participants engage in an activity. Physical activity researchers typically recode time use survey data for physical activity intensity and domain information, a process known as MET-linkage, which has been shown to have good reliability and validity [2, 3]. Data on time spent in specific activities and their contextual dimensions can then be computed [4, 5].

In this issue, three studies demonstrate the merits of secondary analysis of ‘traditional’ time use surveys. Dr. Anne Loyen and colleagues examine the prevalence of domain-specific sedentary behaviours and their sociodemographic correlates using data from the National Dutch Time Use Survey. Their results show the Dutch population spend an average of 8 h of their day in non-occupational sedentary activities; with 90% of leisure time and 70% of travel time spent sedentary. Males, adults aged 18–34 years and 65 years or older, people in full-time employment, and people who are obese are more likely to accumulate high levels of non-occupational sedentary time. In their investigation, Dr. Teresa Harms et al. present cross-country comparisons of daily metabolic expenditures using data from three countries (UK, USA and Poland) in the Harmonised Multinational Time Use Study. Their findings show discretionary physical activity accounts for less than 5% of daily activity, while the rest of the time was divided between sleep, paid work, and unpaid work or leisure time. Harms et al. also observed different patterns in the contribution of different activities to daily energy expenditure by country and demographic characteristics. In their paper, Prof. Michael Bittman and colleagues investigate the social organisation of eating over three decades using multi-national and Australian time use surveys spanning 1974 to 2006. They report the eating patterns of urban Australian adults have changed considerably over three decades with average time spent on eating as the main activity and the number of eating occasions declining; while concurrently, the frequency of eating as a secondary activity that accompanies another task has increased to as much as eating at mealtimes. Overall, urban Australians are spending less time sharing meals with others.

### Innovations in time use methods and data analysis

Growing interest in the study of whole-of-day activity patterns and their associations with health has led to innovations in time use approaches for comprehensive assessment and analysis of the activities of daily living [6]. Here, two studies present new developments in time use methods and measurement. Dr. Teresa Harms and colleagues describe the validation of the Eurostat time use diary using a wearable camera (SensCam) and wrist-worn accelerometer for capturing the activities of daily living with respect to timing, sequence, and duration. Harms and colleagues show that the time use diary methods for studying whole-of-day activity is supported by objective camera and accelerometer comparison data. Dr. Charles Matthews et al. compare four previous-day recall instruments for measuring physical activity and sedentary behaviour in epidemiological studies. They note the major difference between the instruments are in the operationalisation of definitions of sedentary behaviour and light-intensity physical activity, which led to large variations in estimation of time spent physically active and sedentary between instruments.

### The application of time use methods in observational and intervention studies

Time use methods are not limited to population surveillance, but can also be applied in cohort and intervention evaluation studies. In this theme, studies examine the physical activity and change in activity patterns over time of different population subgroups using the Multimedia Activity Recall for Children and Adults [7, 8], a 24-h recall tool. Dr. Sjaan Gomersall et al. report the results of an efficacy trial of a text-message intervention to promote physical activity in people living with and beyond cancer. This mhealth enhanced clinical exercise rehabilitation program for cancer patients led to increases in moderate-to-vigorous physical activity after 4 weeks and reduced sedentary behaviour at 12 weeks. Dr. Tracy Kolbe-Alexander and colleagues examine the movement patterns of shift workers on day shift days, night shift days, and on non-work days. Their results suggest it may be the nature of their work rather than shift work itself that determines shift workers’ physical activity and sedentary behaviour. Finally, Professor Tim Olds et al. report on the 24-h activity compositions of school-aged children during holiday and school periods. Compared to during the school term, children’s holiday activity profiles were characterised by longer sleep, higher TV and video game time, lower vigorous activity, and lower daily energy expenditure. This pattern could lead to significant weight gain if the lower energy expenditure is not compensated for.

### Future directions

Interest in the application of time use methods in public health research has developed in recent years, as demonstrated by the research published in this *BMC Public Health* supplement and elsewhere [9]. Time use approaches offer important insight into physical activity and diet-related behaviours, and importantly the domain, frequency and context of these behaviours, which are not captured by traditional self-report tools or by objective monitoring of physical activity, for example using accelerometry. Time use based instruments also show promise for assessing lower intensity activities of daily living that other physical activity self-report questionnaires omit. This is particularly important, given emerging evidence on the beneficial health impact of light intensity activity [10–12], and the shift to considering the composition of behaviours across 24-h periods [9].

Further, the consideration of all behaviours across a day and their relationships are leading to advancements in analytical methods that aim to take into account the nature of time use data, such as compositional data analysis techniques [6, 13, 14]. These advancements will allow us to further our understanding of how the pattern of time spent in the spectrum of health behaviours impacts health and how to optimise these.

Limitations of the time use approach include a lack of information captured about occupational time, under-reporting of short duration activities (e.g., snacking, napping) and potential misclassification due to diary sensitivity for short duration activities. Nonetheless, with time use surveys having been conducted in many countries for several decades, there is a wealth of data that could be used for public health research.

While traditionally not used in this way, sociological time use surveys have enormous potential to answer research questions related to the public health contexts and, as demonstrated in this supplement, have sufficient methodological rigour to do so. With few population surveillance systems using consistent instruments to assess physical activity over time, and with most systems failing to capture light-intensity physical activity and contextual information, time use surveys provide a non-health focused trove of high-resolution data that can be used to investigate whole-of-day activity patterns. Simultaneously, embracing new time use measurement and analytic approaches will push the boundaries of the field and link traditional methods with innovative ways of thinking about time use and health.
